# *N*-[4-(4-Bromo­phen­yl)thia­zol-2-yl]-4-(piperidin-1-yl)butanamide

**DOI:** 10.1107/S1600536812019204

**Published:** 2012-05-12

**Authors:** Hazem A. Ghabbour, Adnan A. Kadi, Hussein I. El-Subbagh, Tze Shyang Chia, Hoong-Kun Fun

**Affiliations:** a Department of Pharmaceutical Chemistry, College of Pharmacy, King Saud University, PO Box 2457, Riyadh 11451, Saudi Arabia; b Department of Pharmaceutical Chemistry, Faculty of Pharmaceutical Sciences & Pharmaceutical Industries, Future University, Cairo 12311, Egypt; c X-ray Crystallography Unit, School of Physics, Universiti Sains Malaysia, 11800 USM, Penang, Malaysia

## Abstract

In the title compound, C_18_H_22_BrN_3_OS, the piperidine ring adopts a chair conformation. The mean plane of the thia­zole ring forms dihedral angles of 23.97 (10) and 75.82 (10)° with the mean planes of its adjacent benzene and piperidine rings, respectively. An intra­molecular N—H⋯N hydrogen bond generates an *S*(7) ring motif in the mol­ecule. In the crystal, no significant inter­moelcular hydrogen bonds are observed, but a weak π–π inter­action with a centroid–centroid distance of 3.8855 (13) Å occurs.

## Related literature

For the pharmacological activity of 2-amino­thia­zole derivatives, see: Lednicer & Mitscher (1977 ▶); Vagdevi *et al.* (2006 ▶). For ring puckering parameters, see: Cremer & Pople (1975 ▶). For further synthetic details, see: El-Subbagh *et al.* (1999 ▶). For the stability of the temperature controller used in the data collection, see: Cosier & Glazer (1986 ▶).
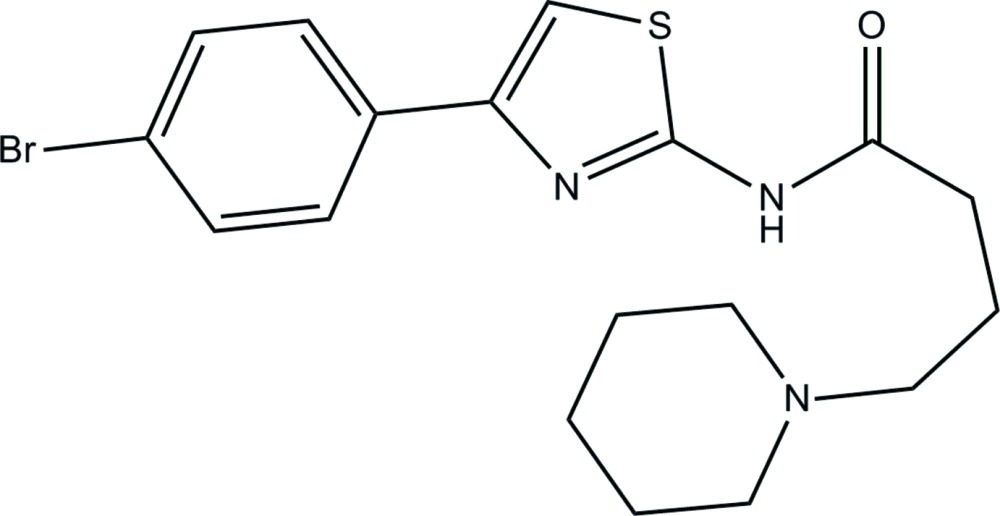


## Experimental

### 

#### Crystal data

C_18_H_22_BrN_3_OS
*M* *_r_* = 408.36
Triclinic, 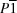

*a* = 6.8276 (7) Å
*b* = 9.2782 (9) Å
*c* = 14.5907 (14) Å
α = 88.812 (2)°
β = 86.085 (3)°
γ = 75.394 (2)°
*V* = 892.33 (15) Å^3^
*Z* = 2
Mo *K*α radiation
μ = 2.43 mm^−1^
*T* = 100 K
0.37 × 0.14 × 0.05 mm

#### Data collection

Bruker APEX DUO CCD area-detector diffractometer
Absorption correction: multi-scan (*SADABS*; Bruker, 2009 ▶) *T*_min_ = 0.467, *T*_max_ = 0.890
17762 measured reflections
5019 independent reflections
4117 reflections with *I* > 2σ(*I*)
*R*_int_ = 0.042

#### Refinement

*R*[*F* ^2^ > 2σ(*F* ^2^)] = 0.042
*wR*(*F* ^2^) = 0.114
*S* = 1.07
5019 reflections
221 parameters
H atoms treated by a mixture of independent and constrained refinement
Δρ_max_ = 1.80 e Å^−3^
Δρ_min_ = −0.86 e Å^−3^

### 

Data collection: *APEX2* (Bruker, 2009 ▶); cell refinement: *SAINT* (Bruker, 2009 ▶); data reduction: *SAINT*; program(s) used to solve structure: *SHELXTL* (Sheldrick, 2008 ▶); program(s) used to refine structure: *SHELXTL*; molecular graphics: *SHELXTL*; software used to prepare material for publication: *SHELXTL* and *PLATON* (Spek, 2009 ▶).

## Supplementary Material

Crystal structure: contains datablock(s) global, I. DOI: 10.1107/S1600536812019204/hb6767sup1.cif

Structure factors: contains datablock(s) I. DOI: 10.1107/S1600536812019204/hb6767Isup2.hkl

Supplementary material file. DOI: 10.1107/S1600536812019204/hb6767Isup3.cml

Additional supplementary materials:  crystallographic information; 3D view; checkCIF report

## Figures and Tables

**Table 1:** Hydrogen-bond geometry (Å, °)

*D*—H⋯*A*	*D*—H	H⋯*A*	*D*⋯*A*	*D*—H⋯*A*
N2—H1*N*2⋯N3	0.93 (3)	1.83 (3)	2.742 (2)	167 (3)

**Fig. 1.**
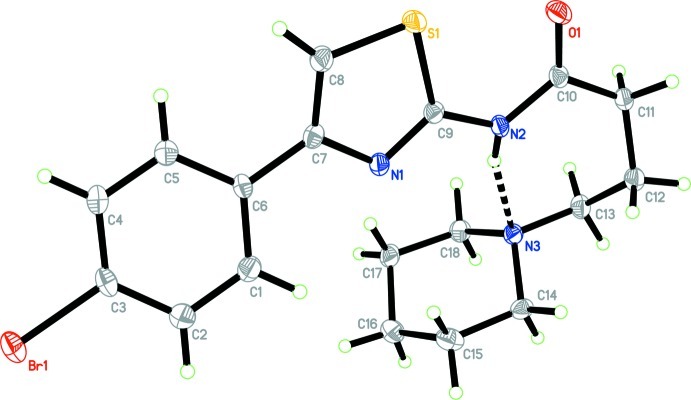
The molecular structure of the title compound with 50% probability displacement ellipsoids. The hydrogen bond is indicated by a dashed line.

## supplementary crystallographic information

### Comment

2-Aminothiazole derivatives possess a wide range of pharmacological activities. Some of them have been used as anti-infective or anti-trichomonal agents. Those, having an aromatic substituent at C-4 position, exhibit some central nervous system (*CNS*) activities (Lednicer & Mitscher, 1977) and have been found to be potent biological response modifiers with significant immunosuppressant activity (Vagdevi *et al.*, 2006).

The asymmetric unit of the title compound is shown in Fig. 1. The piperidine ring (N3/C14–C18) adopts a chair conformation with puckering parameters (Cremer & Pople, 1975), *Q* = 0.572 (2) Å, θ = 2.6 (2)° and φ = 354 (6)°. The atoms N3 and C16 are deviated from the mean plane of C14/C15/C17/C18 by -0.6758 (16) and 0.6567 (18) Å, respectively. The mean plane of central thiazole ring (S1/N1/C7–C9) forms dihedral angles of 23.97 (10) and 75.82 (10)° with the mean planes of adjacent benzene ring (C1–C6) and piperidine ring, respectively. An intramolecular N2—H1N2···N3 hydrogen bond generates an S(7) ring motif in the molecule.

In the crystal, no significant intermoelcular hydrogen bondings are observed. The crystal packing is stabilized by *Cg*3—*Cg*3 interaction with centroid–centroid distance of 3.8855 (13) Å [symmetry code: 1-*X*,1-Y,1-*Z*; *Cg*3 is the centroid of C1–C6 ring].

### Experimental

A mixture of *N*-(4-(4-bromophenyl)thiazol-2-yl)-4-chlorobutanamide (359 mg, 1 mmol) and piperidine (340 mg, 4 mmol) in dry toluene was stirred and heated to reflux. After 12 h, the mixture was cooled down to room temperature and the solvent was removed in vacuum. The residue was purified by chromatotron using CHCl_3_:EtOAc (9:1) as eluting system and the title compound was then crystallized from ethanol (El-Subbagh *et al.*, 1999) as colourless plates.

### Refinement

The atom H1N2 was located from difference fourier map and refined freely [N2—H1N2 = 0.93 (3) Å]. The remaining H atoms were positioned geometrically [C—H = 0.95 and 0.99 Å] and refined using a riding model with *U*_iso_(H) = 1.2 *U*_eq_(C). Twelve outliers (4 5 5), (4 4 5), (4 3 6), (-3 - 2 7), (-3 - 3 7), (4 5 4), (4 4 6), (-2 - 2 5), (4 1 5), (-3 - 2 8), (1 1 2) and (3 1 0) were omitted.

### Crystal data

**Table:**

C_18_H_22_BrN_3_OS	*Z* = 2
*M**_r_* = 408.36	*F*(000) = 420
Triclinic, *P*1	*D*_x_ = 1.520 Mg m^−^^3^
Hall symbol: -P 1	Mo *K*α radiation, λ = 0.71073 Å
*a* = 6.8276 (7) Å	Cell parameters from 5955 reflections
*b* = 9.2782 (9) Å	θ = 3.1–29.8°
*c* = 14.5907 (14) Å	µ = 2.43 mm^−^^1^
α = 88.812 (2)°	*T* = 100 K
β = 86.085 (3)°	Plate, colourless
γ = 75.394 (2)°	0.37 × 0.14 × 0.05 mm
*V* = 892.33 (15) Å^3^	

### Data collection

**Table:**

Bruker APEX DUO CCD area-detector diffractometer	5019 independent reflections
Radiation source: fine-focus sealed tube	4117 reflections with *I* > 2σ(*I*)
Graphite monochromator	*R*_int_ = 0.042
φ and ω scans	θ_max_ = 29.9°, θ_min_ = 1.4°
Absorption correction: multi-scan (*SADABS*; Bruker, 2009)	*h* = −9→9
*T*_min_ = 0.467, *T*_max_ = 0.890	*k* = −12→12
17762 measured reflections	*l* = −20→20

### Refinement

**Table:**

Refinement on *F*^2^	Primary atom site location: structure-invariant direct methods
Least-squares matrix: full	Secondary atom site location: difference Fourier map
*R*[*F*^2^ > 2σ(*F*^2^)] = 0.042	Hydrogen site location: inferred from neighbouring sites
*wR*(*F*^2^) = 0.114	H atoms treated by a mixture of independent and constrained refinement
*S* = 1.07	*w* = 1/[σ^2^(*F*_o_^2^) + (0.0683*P*)^2^ + 0.091*P*] where *P* = (*F*_o_^2^ + 2*F*_c_^2^)/3
5019 reflections	(Δ/σ)_max_ = 0.001
221 parameters	Δρ_max_ = 1.80 e Å^−^^3^
0 restraints	Δρ_min_ = −0.86 e Å^−^^3^

### Special details

**Table:**

Experimental. The crystal was placed in the cold stream of an Oxford Cryosystems Cobra open-flow nitrogen cryostat (Cosier & Glazer, 1986) operating at 100.0 (1) K.
Geometry. All e.s.d.'s (except the e.s.d. in the dihedral angle between two l.s. planes) are estimated using the full covariance matrix. The cell e.s.d.'s are taken into account individually in the estimation of e.s.d.'s in distances, angles and torsion angles; correlations between e.s.d.'s in cell parameters are only used when they are defined by crystal symmetry. An approximate (isotropic) treatment of cell e.s.d.'s is used for estimating e.s.d.'s involving l.s. planes.
Refinement. Refinement of *F*^2^ against ALL reflections. The weighted *R*-factor *wR* and goodness of fit *S* are based on *F*^2^, conventional *R*-factors *R* are based on *F*, with *F* set to zero for negative *F*^2^. The threshold expression of *F*^2^ > σ(*F*^2^) is used only for calculating *R*-factors(gt) *etc*. and is not relevant to the choice of reflections for refinement. *R*-factors based on *F*^2^ are statistically about twice as large as those based on *F*, and *R*- factors based on ALL data will be even larger.

### Fractional atomic coordinates and isotropic or equivalent isotropic displacement parameters (Å^2^)

**Table:**

	*x*	*y*	*z*	*U*_iso_*/*U*_eq_	
Br1	0.82800 (4)	0.81297 (2)	0.549438 (15)	0.02561 (9)	
S1	0.28942 (9)	0.30257 (5)	0.19656 (4)	0.01885 (13)	
O1	−0.0331 (3)	0.31993 (16)	0.09887 (11)	0.0220 (4)	
N1	0.2084 (3)	0.56893 (18)	0.26349 (12)	0.0153 (3)	
N2	−0.0331 (3)	0.54008 (18)	0.16321 (12)	0.0154 (4)	
N3	−0.2166 (3)	0.83694 (18)	0.14310 (12)	0.0154 (4)	
C1	0.3846 (4)	0.6922 (2)	0.40953 (15)	0.0197 (4)	
H1A	0.2429	0.7288	0.4038	0.024*	
C2	0.4825 (4)	0.7643 (2)	0.46664 (15)	0.0221 (5)	
H2A	0.4083	0.8490	0.5007	0.027*	
C3	0.6911 (4)	0.7117 (2)	0.47378 (15)	0.0187 (4)	
C4	0.8022 (4)	0.5877 (2)	0.42470 (14)	0.0191 (4)	
H4A	0.9445	0.5532	0.4294	0.023*	
C5	0.7013 (4)	0.5153 (2)	0.36861 (14)	0.0180 (4)	
H5A	0.7760	0.4298	0.3354	0.022*	
C6	0.4927 (3)	0.5654 (2)	0.35987 (13)	0.0142 (4)	
C7	0.3892 (3)	0.4898 (2)	0.29872 (14)	0.0155 (4)	
C8	0.4539 (4)	0.3451 (2)	0.26968 (15)	0.0197 (4)	
H8A	0.5741	0.2770	0.2876	0.024*	
C9	0.1412 (3)	0.4853 (2)	0.20874 (14)	0.0146 (4)	
C10	−0.1143 (4)	0.4531 (2)	0.11071 (13)	0.0154 (4)	
C11	−0.3088 (4)	0.5259 (2)	0.06591 (15)	0.0177 (4)	
H11A	−0.3945	0.4541	0.0698	0.021*	
H11B	−0.2725	0.5386	−0.0001	0.021*	
C12	−0.4417 (4)	0.6759 (2)	0.10078 (15)	0.0189 (4)	
H12A	−0.5764	0.6917	0.0754	0.023*	
H12B	−0.4621	0.6698	0.1684	0.023*	
C13	−0.3601 (4)	0.8121 (2)	0.07711 (15)	0.0190 (4)	
H13A	−0.2907	0.7993	0.0149	0.023*	
H13B	−0.4754	0.9015	0.0758	0.023*	
C14	−0.3271 (4)	0.9117 (2)	0.22672 (15)	0.0189 (4)	
H14A	−0.4143	0.8503	0.2553	0.023*	
H14B	−0.4158	1.0092	0.2098	0.023*	
C15	−0.1798 (4)	0.9352 (2)	0.29574 (15)	0.0231 (5)	
H15A	−0.1006	0.8373	0.3172	0.028*	
H15B	−0.2573	0.9897	0.3497	0.028*	
C16	−0.0344 (4)	1.0234 (2)	0.25308 (16)	0.0231 (5)	
H16A	−0.1106	1.1268	0.2402	0.028*	
H16B	0.0691	1.0272	0.2967	0.028*	
C17	0.0692 (4)	0.9490 (2)	0.16389 (16)	0.0211 (5)	
H17A	0.1518	1.0120	0.1332	0.025*	
H17B	0.1613	0.8515	0.1782	0.025*	
C18	−0.0854 (4)	0.9263 (2)	0.09965 (15)	0.0195 (4)	
H18A	−0.1701	1.0244	0.0814	0.023*	
H18B	−0.0140	0.8754	0.0434	0.023*	
H1N2	−0.083 (5)	0.643 (3)	0.163 (2)	0.031 (8)*	

### Atomic displacement parameters (Å^2^)

**Table:**

	*U*^11^	*U*^22^	*U*^33^	*U*^12^	*U*^13^	*U*^23^
Br1	0.02680 (16)	0.02875 (14)	0.02536 (13)	−0.01282 (10)	−0.00715 (10)	−0.00372 (9)
S1	0.0190 (3)	0.0121 (2)	0.0252 (3)	−0.0019 (2)	−0.0067 (2)	−0.00195 (18)
O1	0.0235 (9)	0.0153 (7)	0.0278 (8)	−0.0044 (7)	−0.0060 (7)	−0.0037 (6)
N1	0.0127 (9)	0.0143 (7)	0.0193 (8)	−0.0041 (7)	−0.0024 (7)	−0.0009 (6)
N2	0.0123 (9)	0.0126 (7)	0.0220 (8)	−0.0037 (7)	−0.0043 (7)	−0.0018 (6)
N3	0.0147 (9)	0.0136 (7)	0.0187 (8)	−0.0050 (7)	−0.0002 (7)	−0.0003 (6)
C1	0.0156 (11)	0.0191 (9)	0.0237 (10)	−0.0031 (9)	−0.0020 (8)	−0.0008 (8)
C2	0.0205 (12)	0.0209 (9)	0.0248 (10)	−0.0050 (9)	−0.0012 (9)	−0.0036 (8)
C3	0.0215 (12)	0.0210 (9)	0.0175 (9)	−0.0117 (9)	−0.0053 (8)	0.0019 (7)
C4	0.0167 (11)	0.0223 (9)	0.0199 (9)	−0.0070 (9)	−0.0053 (8)	0.0029 (8)
C5	0.0172 (11)	0.0184 (9)	0.0187 (9)	−0.0049 (8)	−0.0020 (8)	0.0003 (7)
C6	0.0145 (10)	0.0143 (8)	0.0156 (8)	−0.0062 (8)	−0.0038 (8)	0.0018 (7)
C7	0.0125 (10)	0.0161 (8)	0.0181 (9)	−0.0043 (8)	−0.0004 (8)	0.0022 (7)
C8	0.0170 (12)	0.0169 (9)	0.0247 (10)	−0.0022 (8)	−0.0061 (9)	0.0004 (7)
C9	0.0133 (10)	0.0131 (8)	0.0178 (8)	−0.0044 (8)	−0.0001 (8)	0.0008 (6)
C10	0.0151 (11)	0.0169 (8)	0.0160 (8)	−0.0076 (8)	0.0003 (8)	0.0000 (7)
C11	0.0147 (11)	0.0174 (9)	0.0225 (9)	−0.0064 (8)	−0.0031 (8)	−0.0014 (7)
C12	0.0158 (11)	0.0190 (9)	0.0222 (10)	−0.0043 (8)	−0.0036 (8)	−0.0011 (7)
C13	0.0168 (11)	0.0171 (9)	0.0240 (10)	−0.0050 (8)	−0.0053 (9)	0.0017 (7)
C14	0.0178 (11)	0.0163 (9)	0.0226 (10)	−0.0061 (8)	0.0050 (9)	−0.0026 (7)
C15	0.0300 (14)	0.0195 (9)	0.0209 (10)	−0.0090 (10)	0.0031 (9)	−0.0032 (8)
C16	0.0243 (13)	0.0198 (9)	0.0269 (11)	−0.0087 (9)	−0.0015 (9)	−0.0039 (8)
C17	0.0156 (11)	0.0171 (9)	0.0322 (11)	−0.0075 (8)	0.0013 (9)	−0.0029 (8)
C18	0.0193 (12)	0.0190 (9)	0.0218 (9)	−0.0088 (9)	0.0022 (8)	−0.0003 (7)

### Geometric parameters (Å, º)

**Table:**

Br1—C3	1.898 (2)	C8—H8A	0.9500
S1—C8	1.720 (2)	C10—C11	1.515 (3)
S1—C9	1.7473 (19)	C11—C12	1.533 (3)
O1—C10	1.231 (2)	C11—H11A	0.9900
N1—C9	1.306 (3)	C11—H11B	0.9900
N1—C7	1.393 (3)	C12—C13	1.529 (3)
N2—C10	1.362 (3)	C12—H12A	0.9900
N2—C9	1.380 (3)	C12—H12B	0.9900
N2—H1N2	0.93 (3)	C13—H13A	0.9900
N3—C18	1.472 (3)	C13—H13B	0.9900
N3—C14	1.478 (3)	C14—C15	1.527 (4)
N3—C13	1.480 (3)	C14—H14A	0.9900
C1—C2	1.383 (3)	C14—H14B	0.9900
C1—C6	1.407 (3)	C15—C16	1.532 (4)
C1—H1A	0.9500	C15—H15A	0.9900
C2—C3	1.394 (4)	C15—H15B	0.9900
C2—H2A	0.9500	C16—C17	1.528 (3)
C3—C4	1.390 (3)	C16—H16A	0.9900
C4—C5	1.389 (3)	C16—H16B	0.9900
C4—H4A	0.9500	C17—C18	1.514 (3)
C5—C6	1.396 (3)	C17—H17A	0.9900
C5—H5A	0.9500	C17—H17B	0.9900
C6—C7	1.466 (3)	C18—H18A	0.9900
C7—C8	1.369 (3)	C18—H18B	0.9900
			
C8—S1—C9	88.45 (10)	H11A—C11—H11B	106.9
C9—N1—C7	110.70 (17)	C13—C12—C11	116.0 (2)
C10—N2—C9	122.88 (17)	C13—C12—H12A	108.3
C10—N2—H1N2	121 (2)	C11—C12—H12A	108.3
C9—N2—H1N2	116 (2)	C13—C12—H12B	108.3
C18—N3—C14	110.36 (17)	C11—C12—H12B	108.3
C18—N3—C13	109.99 (17)	H12A—C12—H12B	107.4
C14—N3—C13	110.74 (18)	N3—C13—C12	113.05 (17)
C2—C1—C6	120.7 (2)	N3—C13—H13A	109.0
C2—C1—H1A	119.7	C12—C13—H13A	109.0
C6—C1—H1A	119.7	N3—C13—H13B	109.0
C1—C2—C3	119.5 (2)	C12—C13—H13B	109.0
C1—C2—H2A	120.3	H13A—C13—H13B	107.8
C3—C2—H2A	120.3	N3—C14—C15	110.96 (19)
C4—C3—C2	121.1 (2)	N3—C14—H14A	109.4
C4—C3—Br1	119.07 (19)	C15—C14—H14A	109.4
C2—C3—Br1	119.76 (17)	N3—C14—H14B	109.4
C5—C4—C3	118.7 (2)	C15—C14—H14B	109.4
C5—C4—H4A	120.6	H14A—C14—H14B	108.0
C3—C4—H4A	120.6	C14—C15—C16	111.38 (19)
C4—C5—C6	121.5 (2)	C14—C15—H15A	109.4
C4—C5—H5A	119.2	C16—C15—H15A	109.4
C6—C5—H5A	119.2	C14—C15—H15B	109.4
C5—C6—C1	118.5 (2)	C16—C15—H15B	109.4
C5—C6—C7	120.87 (18)	H15A—C15—H15B	108.0
C1—C6—C7	120.6 (2)	C17—C16—C15	109.71 (19)
C8—C7—N1	114.3 (2)	C17—C16—H16A	109.7
C8—C7—C6	126.45 (19)	C15—C16—H16A	109.7
N1—C7—C6	119.23 (17)	C17—C16—H16B	109.7
C7—C8—S1	111.31 (17)	C15—C16—H16B	109.7
C7—C8—H8A	124.3	H16A—C16—H16B	108.2
S1—C8—H8A	124.3	C18—C17—C16	111.1 (2)
N1—C9—N2	121.60 (17)	C18—C17—H17A	109.4
N1—C9—S1	115.24 (16)	C16—C17—H17A	109.4
N2—C9—S1	123.16 (15)	C18—C17—H17B	109.4
O1—C10—N2	121.7 (2)	C16—C17—H17B	109.4
O1—C10—C11	120.4 (2)	H17A—C17—H17B	108.0
N2—C10—C11	117.80 (17)	N3—C18—C17	111.59 (18)
C10—C11—C12	120.40 (18)	N3—C18—H18A	109.3
C10—C11—H11A	107.2	C17—C18—H18A	109.3
C12—C11—H11A	107.2	N3—C18—H18B	109.3
C10—C11—H11B	107.2	C17—C18—H18B	109.3
C12—C11—H11B	107.2	H18A—C18—H18B	108.0
			
C6—C1—C2—C3	1.0 (3)	C10—N2—C9—N1	−175.4 (2)
C1—C2—C3—C4	−0.2 (3)	C10—N2—C9—S1	5.7 (3)
C1—C2—C3—Br1	178.34 (17)	C8—S1—C9—N1	−0.78 (18)
C2—C3—C4—C5	−0.7 (3)	C8—S1—C9—N2	178.24 (19)
Br1—C3—C4—C5	−179.21 (16)	C9—N2—C10—O1	−1.9 (3)
C3—C4—C5—C6	0.8 (3)	C9—N2—C10—C11	178.70 (19)
C4—C5—C6—C1	0.0 (3)	O1—C10—C11—C12	162.6 (2)
C4—C5—C6—C7	178.56 (19)	N2—C10—C11—C12	−18.0 (3)
C2—C1—C6—C5	−0.9 (3)	C10—C11—C12—C13	73.2 (3)
C2—C1—C6—C7	−179.45 (19)	C18—N3—C13—C12	157.54 (18)
C9—N1—C7—C8	−0.6 (3)	C14—N3—C13—C12	−80.2 (2)
C9—N1—C7—C6	178.28 (19)	C11—C12—C13—N3	−84.7 (2)
C5—C6—C7—C8	23.5 (3)	C18—N3—C14—C15	−58.9 (2)
C1—C6—C7—C8	−158.0 (2)	C13—N3—C14—C15	179.05 (17)
C5—C6—C7—N1	−155.2 (2)	N3—C14—C15—C16	56.3 (2)
C1—C6—C7—N1	23.3 (3)	C14—C15—C16—C17	−52.9 (3)
N1—C7—C8—S1	0.0 (3)	C15—C16—C17—C18	53.2 (2)
C6—C7—C8—S1	−178.77 (17)	C14—N3—C18—C17	59.7 (2)
C9—S1—C8—C7	0.39 (18)	C13—N3—C18—C17	−177.78 (17)
C7—N1—C9—N2	−178.11 (19)	C16—C17—C18—N3	−57.3 (2)
C7—N1—C9—S1	0.9 (2)		

### Hydrogen-bond geometry (Å, º)

**Table:**

*D*—H···*A*	*D*—H	H···*A*	*D*···*A*	*D*—H···*A*
N2—H1*N*2···N3	0.93 (3)	1.83 (3)	2.742 (2)	167 (3)
